# Scratch2, a Snail Superfamily Member, Is Regulated by miR-125b

**DOI:** 10.3389/fcell.2020.00769

**Published:** 2020-08-25

**Authors:** Carolina Purcell Goes, Felipe Monteleone Vieceli, Shirley Mirna De La Cruz, Marcos Simões-Costa, Chao Yun Irene Yan

**Affiliations:** ^1^Department of Cell and Developmental Biology, Institute of Biomedical Sciences, University of São Paulo, São Paulo, Brazil; ^2^Department of Molecular Biology and Genetics, College of Agriculture and Life Sciences, Cornell University, Ithaca, NY, United States

**Keywords:** Scratch2, Snail superfamily, miRNA, neural tube, CRISPR/Cas9

## Abstract

*Scratch2* is a transcription factor expressed in a very restricted population of vertebrate embryonic neural cell precursors involved in their survival, differentiation, and migration. The mechanisms that control its expression remain unknown and could contribute towards our understanding of gene regulation during neural differentiation and evolution. Here we investigate the role of microRNAs (miRNAs) in the Scrt2 post-transcriptional regulatory mechanism. We identified binding sites for miR-125b and -200b in the Scrt2 3′UTR in silico. We confirmed the repressive-mediated activity of the Scrt2 3′UTR through electroporation of luciferase constructs into chick embryos. Further, both CRISPR/Cas9-mediated deletion of miR-125b/-200b responsive elements from chicken Scrt2 3′UTR and expression of miRNAs sponges increased Scrt2 expression field, suggesting a role for these miRNAs as post-transcriptional regulators of Scrt2. The biological effect of miR-125b titration was much more pronounced than that of miR-200b. Therefore, we propose that, after transcription, miR-125b fine-tunes the Scrt2 expression domain.

## Introduction

Neurodifferentiation in the embryonic neural tube is orchestrated by the spatially and temporally restricted expression of transcription factors (TF). The Scratch family, part of the SNAIL superfamily of zinc-finger TFs, has a conserved role in neural development. In the early stages of neural differentiation of distant animal species, Scratch2 (Scrt2) is expressed in early post-mitotic cells initiating neural differentiation and migration (Ellis and Horvitz, 1991; Roark et al., 1995; Marín and Nieto, 2006; Dam et al., 2011; Rodríguez-Aznar and Nieto, 2011; Paul et al., 2012; Itoh et al., 2013; Vieceli et al., 2013). The precise boundaries of Scrt2 expression suggests the existence of mechanisms for tight regulation of transcript expression and availability. Nevertheless, nothing is known about elements with post-transcriptional regulation activity on Scrt2 expression.

MicroRNAs (miRNAs), a class of small non-coding RNAs, mediate a mechanism of post-transcriptional regulation in which they interact with target sequences at the 3′UTR region of mRNAs to inhibit translation or induct transcript degradation (reviewed by Bartel, 2004). There is growing evidence of the importance of miRNAs in neural development, including cell survival, proliferation and migration (Bak et al., 2008; Murphy et al., 2010; Renthal et al., 2010; Lim and Thiery, 2012; Ding et al., 2013; Yao et al., 2013; Hong et al., 2017; Clark et al., 2018). Indeed, previous studies have shown evidence of miRNAs directly repressing function of SNAIL superfamily members Snail1 and Snail2/Slug during development, as well as in several types of cancer (reviewed by Zhou et al., 2019). For instance, miRNAs miR-1, miR-124, and of the miR-200b family were all associated with reduction of metastasis aggressiveness and epithelial to mesenchymal transition (EMT) impairment through Snail2 repression (Ambs et al., 2008; Burk et al., 2008; Davalos et al., 2012; Liang et al., 2013; Liu Y. N. et al., 2013; Perdigão-Henriques et al., 2016). Also, miR-124a, miR-30, and miR-206 were all shown to repress Snail1/2 during myoblast differentiation and gastrulation in human embryoid bodies (Lee et al., 2010; Soleimani et al., 2012).

Given the evidence for a role of miRNAs in repressing expression of SNAIL TFs, we investigated how miRNAs control Scrt2 expression in the neural tube of chick embryos. We first looked for miRNA responsive elements (MRE) in Scrt2 orthologs and identified miR-125b and -200b as putative candidates based on evolutionary conservation. We also employed an *in vivo* luciferase reporter assay to determine whether the presence of MREs in cScrt2 3′UTR can mediate post-transcriptional repression. Finally, we looked at the effect of ablating miR-125b/-200b interaction with Scrt2 – using CRISPR/Cas9 and miRNA sponges – on its final expression pattern in the posterior neural tube.

## Materials and Methods

### Identification of Candidate miRNAs Responsive Elements

Conserved miRNA targets in the chicken Scrt2 3′UTR (ENST00000246104.6) were searched using *TargetScan v7.2* (Agarwal et al., 2015^1^. An overlapping site for miR-125b and miR-200bc/-429 and another one for miR-204/-211 were identified and the expression pattern of these miRNAs was verified at GEISHA (*Gallus* Expression *in situ* Hybridization Analysis, Darnell et al., 2006)^2^. Only miR-125b and miR-200b were expressed in the neural tube.

Corresponding regions of the chicken genome (galGal6) miR-125/-200b overlapping site in other species were retrieved from the UCSC Genome Browser multiple alignment of 77 vertebrates. The resulting alignment showed conservation among amniotes only. To confirm the candidate homologous elements in *X. tropicalis* and zebrafish Scrt2, their predicted cDNA sequences were scanned for miR-125b/-200b sites and sequences surrounding the candidates identified were aligned to human, mouse, chicken and painted turtle REs obtained previously. The zebrafish candidates were also identified using TargetScan. The phylogenetic tree was constructed based on multiple alignment using PhyloP (UCSC Genomics; Siepel et al., 2005) and edited on iTOL v4 (Letunic and Bork, 2019)^3^ to propose the appearance of miR-125b and 200b responsive elements during the evolution of Scrt2 (Supplementary Figure S1).

### Cloning

To clone the 3′UTR region of Scrt2 for the *in vivo* luciferase assay, we designed primers based on a 521-bp region of the putative chicken Scrt2 3′UTR generated by 3P-seq tags (Jan et al., 2011). The region was amplified by PCR of cDNA from HH23 embryos. The primers (F- 5′CTCGAGACCGGAGGCGGATCGCCGTGC and R- 5′ TCTAGATAGTGGCAGAAGTCCCTTTTATA) contained XhoI and XbaI restriction sites at their 5′ ends. The product was cloned into pGEM-T Vector (Promega) and subcloned downstream of the luciferase coding region in the pmiR-Glo vector (Promega). The resulting plasmid was named pmiR-GLO-cScrt2UTR.

For depleting miRNAs *in vivo*, we designed and ordered sponges sequences (GenScript, United States)^4^ containing seven MREs in tandem for each miRNA, flanked by restriction sites for XbaI and PmeI for subsequent subcloning into the pRNA-U6.1 vector (Addgene # 35664). The sponge sequence for miR-125b was 5′- TCACAAGTTACCACTCAGGGACGATTCACAAGTTACCAC TCAGGGAACCGGTTCACAAGTTACCACTCAGGGATCACT CACAAGTTACCACTCAGGGATCACAAGTTACCACCGATT CAGGGACGATTAG and for miR-200b was 5′- TCACAAGTTACCACCAGTATTACGATTCACAAGTTACCAC CAGTATTAACCGGTTCACAAGTTACCACCAGTATTATCAC TCACAAGTTACCACCAGTATTATCACAAGTTACCACCGAT CAGTATTACGATTAG.

For CRISPR/Cas9 genome editing, the sgRNA was designed to target the miR-125b site within the cScrt2 3′-UTR and annealed from single stranded oligos (sgRNA-F 5′- AGTCGTGCAATTCAGGGATATAAAA; sgRNA-R 5′- AAACTTTTATATCCCTGAATTGCAC) designed to form overhangs compatible with pcU6.3-sgRNA vector digested with BsmBI (NEB, cat. R0580S). The oligos were cloned into the pcU6.3-sgRNA vector as described (Williams et al., 2018). A scrambled control guide was generated using the “RNA sequence scrambler” tool (GenScript, United States)^4^, with the chicken genome as a base for the search of off-targets, and cloned from the oligos sgRNA Scrambled-F 5′-AGTCGGCAGGAATCAATTAGATAAT and sgRNA Scrambled-R 5′-AAACATTATCTAATTGATTCCTGCC). All final clones were sequenced to ensure that the cloned guide had no mismatches.

### Chicken Embryos

Fertilized eggs from *Gallus gallus* Leghorn (Yamaguishi Farm, São Paulo, Brazil) or White Leghorn hens (University of Connecticut, Department of Animal Science – United States) were incubated at 37.8°C and 50% humidity until the desired developmental stages according to Hamburger and Hamilton (Hamburger and Hamilton, 1992). All procedures were approved by our institutional ethic committees (CEUA ICB/USP n° 025/2013).

### *In ovo* Electroporation

Electroporation procedures followed standard protocols (Harada et al., 2017). The plasmids pmiR-GLO-cScrt2UTR, pmiR-GLO, pRNA-6.1-125b-Sponge or pRNA-6.1-200b-Sponge (3 μg/μL) were mixed with pCDNA3.1-mGFP (2 μg/μL), together with 0.2% Fast Green dye (Sigma Aldrich, United States). For CRISPR/Cas9 experiments, pCAG-Cas9-2A-Citrine was mixed with pcU6.3-3′UTR-sgRNA or pcU6.3-3′UTR-sgRNA-Scrambled. Electroporation parameter were: 5 pulses of 20 V, 50 ms of duration and 100 ms of interval. Embryos were reincubated, screened for successful transfection, collected at stage HH23 and processed further accordingly.

### Luciferase Assay

Forty-eight hours post-electroporation, the neural tube of HH23 embryos were collected in Ringer′s solution. Three control and three experimental halves were collected and matched by electroporation extension and GFP intensity. Each sample was lysed in 1× lysis buffer from Dual-Luciferase Reporter Assay System kit (Promega, cat. #E1910) and luciferase activity detection was performed according to manufacturer’s instructions in a Synergy HT luminometer (Biotek, United States). Three technical triplicates were read for each biological sample.

### Embryo Dissociation and Cell Sorting (FACS)

Neural tube halves of HH23 embryos unilaterally electroporated with CRISPR/Cas9 system were individually microdissected with a tungsten needle (0,125 mm) and kept in Ringer’s solution until dissociation in Accumax (Accutase cat. #SCR006) cell dissociation solution for 40 min at room temperature under mild agitation. After dissociation, cells were passed through a 40 μm cell strainer (Pluriselect USA, Mini Cell Strainer II, 45-09840-50) and centrifuged at 400 g for 10 min. The supernatant was carefully discarded and cells were resuspended in 200 ml of Hank’s Balanced Salt Solution (HBSS) with 50 mM EDTA, 100 mM HEPES, pH 8.0 and 0.5% BSA. At least 4000 Citrine-positive or negative cells were sorted through fluorescence-activated cell sorting (FACS) process from experimental or control side, respectively, directly into 50 μL of lysis buffer from Power SYBR Green Cells-to-CT kit (Thermo Fisher Scientific, 4402953) using BD AriaFusion cell sorter. Control-side cells were randomly selected to match the number of contralateral citrine-positive cells.

### RT-qPCR

For miRNAs absolute quantification, the truncal portions of three neural tubes from HH22 embryos were microdissected and pooled for lysis and RNA isolation with TRIzol (Invitrogen, United States). For miRNA cDNA synthesis, we used the Taqman miRNA Assay kit (Applied Biosystems, United States) with sequence-specific primers for miR-125b and miR-200b and 24 ng total RNA as input. For quantification, we used Taqman probes (Taqman miRNA Assay; Applied Biosystems) to detect hsa-miR-125b (ID 000449) and gga-miR-200b (ID 006005). We performed the quantification in three technical replicates for each sample with 6 ng cDNA per reaction. The mimics of both miRNAs (miRVANA hsa-miR-125b-5p, cat. MC10148 and gga-miR-200b-3p, cat MC1050 – Thermo Fisher Scientific, EUA) were used to generate a standard curve with serial dilutions from 7.2 to 000.72 μM/μL.

For data acquisition and analysis, we used the QuantStudio 12K Flex Real-Time PCR System (Applied Biosystems, United States) equipment. The absolute quantification was analyzed by the QuantStudio 12K software (v1.4).

For Scrt2 quantification in CRISPR-edited neural tubes, cDNA was synthesized from FACS-sorted samples with a cell-to-cT kit (Invitrogen, United States). The quantification was performed with SyBr Green (Applied Biosciences, cat. 4368577) in technical triplicates on the ViiA 7 Real-Time PCR System (Applied Biosystems, United States). The primers used were *cScrt2-*F 5′ CTGCTGCAGGGCCACATGCGTTCGCACA and *cScrt2-*R 5′ GCACTGCTTGCACTTGTAGTGCTT. HPRT was used as an endogenous control and detected with the primers *cHPRT-F* 5′ TGGTGAAAGTGGCCAGTTTG and *cHPRT-R* 5′ TCATTGTAGTCGAGGGCGTATC.

Expression relative to loading controls was calculated using the 2-ΔΔCt method (Livak and Schmittgen, 2001).

### CRISPR/Cas9 Editing Validation

**FIGURE 2:**
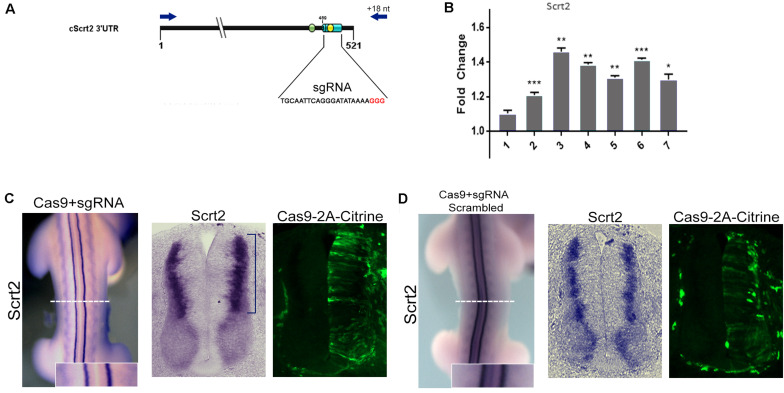
Genomic removal of cScrt2 3’-UTR MRE increase cScrt2 levels. **(A)** Diagram indicating the location of the sgRNA target (light blue cylinder) overlapping the miR-125b site (yellow circle) in the cScrt2 3’UTR. Red nucleotides indicate the PAM sequence. The green circle indicates the position of the miR-200b site. The dark blue arrows indicate the primers positions used for the 3’UTR genomic PCR. **(B)** RT-qPCR for cScrt2 in dissected neural tube from seven different embryos after sgRNA electroporation. The graph depicts the normalized fold change expression relative to the control side of the neural tube. Each bar is a different embryo. Unpaired Student *t*-test, *p < 0.05, **p < 0.01, ***p < 0.001. **(C)** Dorsal view and cross section of an electroporated embryo with Cas9-sgRNA in the right side of the neural tube. The blue bracket indicates the increase in cScrt2 expression domain as compared to the control left neural tube (*n* = 15). **(D)** Dorsal view and cross section of an embryo electroporated with scrambled Cas9-sgRNA in the right side of the neural tube (*n* = 3). Cas9-positive cells are identified as citrine-positive cells through immunofluorescence. The inset is a higher magnification of the white dashed line region.

After unilateral electroporation of Cas9 and UTR-sgRNA, the electroporated side of three neural tubes were dissected independently and dissociated as described above. We then performed FACS to select at least 3000 Citrine-positive cells from each neural tube. Genomic DNA (gDNA) was extracted with TRIzol (Invitrogen, United States) and total RNA with Arcturus PicoPure RNA Isolation Kit (Thermo Fisher Scientific, United States). The gDNA edited region was PCR-amplified (F – 5′ ACCGGAGGCGGATCGCCGTGC and R – 5′ CCACCGCCGCGTGCACAAACA, Figure 2A), gel-purified and cloned into pGEM-T vector (Promega, United States). Thirty-five positive clones were Sanger sequenced to verify successful edition of the target region. The sequences were aligned in UniPro uGene software (v1.29.0) (Supplementary Figure S2). For evaluation of CRISPR-edited transcripts, the total RNA was first converted into cDNA with SuperScript IV RT (Invitrogen, United States, cat. 18090200) and a 250 bp fragment including the sgRNA target region was PCR-amplified with primers containing overhang adapter sequences (F – 5′ TCGTCGGCAGCGTCAGATGTGTATAAGAGACAGAGGGTC ACTTTGAGCCCCGTG and R – 5′ GTCTCGTGGGCTCGGA GATGTGTATAAGAGACAGCTGGTAGTGGCAGAAGTCCC). The products were gel-separated and purified using Wizard SV Gel and PCR Clean-Up System (Promega, United States). The libraries were prepared with Nextera XT Index kit v2 (Illumina, United States) and deep-sequenced using paired-end reads (2 × 250 bp) in Illumina MiSeq System equipment at the Laboratory of Animal Biotechnology (ESALQ/University of São Paulo, Brazil). FastQ reads were submitted to quality analysis and mapped to the cScrt2 3′UTR in galGal5 reference genome using BWA v. 0.7.17 (Burrows-Wheeler Aligner; Li and Durbin, 2009). Transcript variants were determined using the R package CrispRVariants v.1.16.0 (Lindsay et al., 2016).

### *In situ* Hybridization and Immunohistochemistry

*In situ* hybridization was performed on stage HH22-23 embryos post-electroporation of miRNA sponges or CRISPR/Cas9 plasmids, as previously described (Acloque et al., 2008). The whole mount embryos were imaged with Nikon SMZ1500 stereomicroscope and then processed for gelatin-sucrose embedding (Bronner-Fraser et al., 1996). Cross sections with 25 μm of trunk neural tube were collected by cryosectioning (Fisher Scientifics, Waltham, MA, United States).

For immunohistochemistry, we used a rabbit IgG anti-GFP antibody (1:200, Molecular Probes, cat. A-6455) and Alexa 488 goat anti-rabbit IgG antibody (1:400, Molecular Probes, cat. A-11008). The anti-GFP antibody was used to detect the expression of citrine (Nagai et al., 2002). Stained sections were mounted in FluoroShield Mounting (Abcam, United States) and imaged with Zeiss Axio Imager.D2 coupled with an Axiocam 503 Color camera.

### Statistics

We used GraphPad Prism v.7 for statistical calculations. For 3′-UTR luciferase reporter assay, three control and three experimental embryos were assayed as technical triplicates and analyzed with unpaired Student’s *t*-test where *p* < 0.05 was considered as significant. For cScrt2 detection post-CRISPR/Cas9 editing, seven embryos were analyzed and the results were statistically evaluated with Student’s *t*-test.

## Results

### Scrt2 3′UTR Contains miRNA Responsive Elements That Modulate Transcript Availability *in embrio*

**FIGURE 1:**
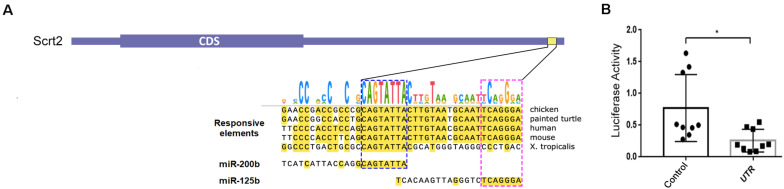
Chicken Scratch2 3′UTR has responsive elements for miR-125b/-200b and regulatory activity in the neural tube. **(A)** Diagram showing the architecture of cScrt2 transcript. The 3’UTR (*galGal6*) alignment of an element conserved across tetrapods that bears a RE (yellow horizontal box; galGal6 chr20:9,921,503–9,921,544) for miR-200b and miR-125b. The seed sequence for miR-200b (GUCAUAAU, blue dashed box) is located at 49 nt and for miR-125b (AGUCCCU, pink dashed box) at 19 nt upstream of the predicted polyadenylation site. **(B)** In embryo luciferase activity in the absence (Control) and presence of cScrt2 3’UTR (UTR) downstream of the luciferase coding region. *n* = 3, *p* < 0.05.

To identify functional MREs in Scrt2 genes, we searched conserved responsive elements (REs) in the 3′UTRs of vertebrates. Despite the 3′UTRs sequence variations, we found an element containing overlapping REs for miR-125b and miR-200b that was conserved among amniotes (Figure 1A). The conservation of these overlapping sites in amniotes and the presence of miR-200b sites in orthologs of other jawed vertebrates suggests biological significance.

Most miRNAs repress gene function by binding to a specific sequence at the 3′UTR of a target mRNA to cause transcript degradation. Thus, we verified the ability of cScrt2 3′UTR to mediate modulation of mRNA levels by electroporating either a reporter chimera, where this portion of the UTR is downstream of luciferase (pmiR-GLO-cScrt2UTR), or a control plasmid (pmiR-GLO), into the neural tube of HH12 embryos. Both plasmids were co-electroporated with membrane GFP (pCDNA3.1-mGFP) to assess electroporation efficacy prior to tissue lysis. As shown in Figure 1B, our results indicated that cScrt2 3′UTR reduced luciferase production to 32% (a 3-fold reduction; *p* < 0.05) of levels produced in control embryos.

### Ablation of miRNA Activity Increases the Levels and Expression Field of cScrt2

To investigate how miR-125b and -200b might affect endogenous cScrt2 transcripts, we looked at the effect of deleting their MREs in the cScrt2 3′UTR genomic region with CRISPR/Cas9 (Figure 2A). A scrambled sgRNA was used as control and edition by our MRE-targeted guide was verified by sequencing (Supplementary Figure S2). The transfected cells from the experimental and control neural tube sides of single HH23 embryos were isolated by FACS and analyzed with RT-qPCR. Six of seven embryos analyzed revealed a significant increase in Scrt2 levels (Figure 2B), suggesting that the REs for miR-125b and miR-200b can regulate cScrt2 levels post-transcriptionally. Deletion of these MREs with CRISPR/Cas9 also expanded the cScrt2 expression field in the neural tube (Figure 2C), phenotype observed both macroscopically (in the whole embryo – Figure 2C) and in histological sections, more pronouncedly in the dorsal domain. Electroporation of scrambled sgRNA elicited no phenotype (Figure 2D). Moreover, ablation of the MREs also disrupted the centrifugal movement of the electroporated cells, which became distributed throughout the center-peripheric layers of the neural tube. In control neural tubes, the electroporated cells were located toward the outer layers of the neural tube.

We also reduced miRNA availability with miRNA sponge constructs. These constructs produce RNAs that contain tandemly repeated MREs to miR-125b or miR-200b (Ebert and Sharp, 2010), to sequester each endogenous miRNA specifically and decrease their availability to act on endogenous targets, including cScrt2.

**FIGURE 3:**
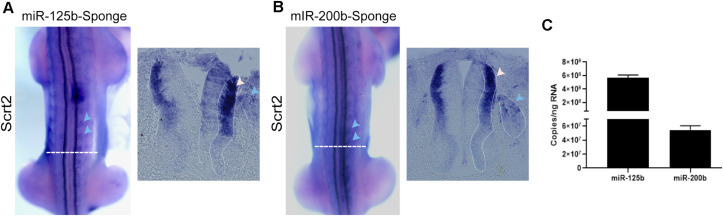
Reduction of miRNA availability increases cScrt2 expression. Dorsal view and cross sections of HH22-23 embryos electroporated with sponge for **(A)** miR-125b (*n* = 12) and **(B)** miR-200b (*n* = 11) in the right side of the neural tube. The horizontal dashed white lines indicate the position of the cross section. The blue and white arrows in **(A,B)** highlight the increase in cScrt2 expression field compared to the contralateral side. The dashed line pointed by the blue arrow shows the perimeter of the DRGs and the dashed line in the neural tube delimits the intermediate zone (IZ). **(C)** Absolute copy number of miR-125b and miR-200b per ng of total RNA in dissected neural tubes dissected from HH22 embryos. The standard curves of miR-125b and -200b showed slopes of -4.19 and -3.71, respectively. The regression coefficient (*r*^2^) of the miR-125b standard curve was 0.998, and PCR efficiency was 73%. For miR-200b, the *r*^2^ was 0.989 and PCR efficiency was 85%.

The miR-125b sponge caused a pronounced increase in the cScrt2 expression field (Figure 3A), such that transcripts were no longer restricted to the intermediate zone (IZ), and there was an increase in expression levels in dorsal root ganglia (DRG). The miR-200b sponge increased cScrt2 expression in the DRG in a similar manner, but only slightly expanded the neural tube expression field (Figure 3B). The efficiency of miRNA titration depends on both the abundance of the target and the endogenous miRNA levels (Mukherji et al., 2011). Thus, we hypothesized that the difference in sponge-generated phenotype could be the result of differences in miRNA levels. Our results show that the absolute copy number for miR-125b was 10-fold higher (5.67 × 10^8^) than miR-200b (5.44 × 10^7^) in the neural tube of HH22 embryos (Figure 3C).

Due to the close proximity of miR-125b and -200b REs (14 bp apart) and the unpredictable outcome associated with CRISPR/Cas9-mediated NHEJ (Williams et al., 2018), we could not guarantee that one sgRNA would only target one of the REs singly. Although our sgRNA target was centered at the miR-125b binding site (Figure 2A), our gDNA sequencing results showed that, in several events, both MREs were edited (Supplementary Figure S2).

**FIGURE 4:**
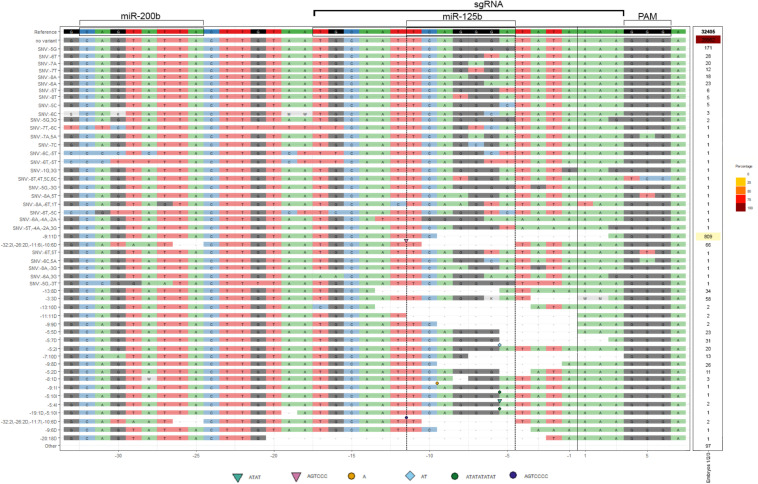
cScrt2 3’UTR CRISPR-Cas9-mediated edition often inactivates the miR-125b RE. The deep-sequencing data obtained from three neural tubes were combined after alignment and sorted by their edition profile from single nucleotide variants (SNV) to InDels (I for insertion and D for deletion). The geometric symbols indicate the position and the nucleotide sequence inserted. The most prevalent edition was the deletion of 11 nucleotides upstream (-9:11D) of the cut site (black vertical line). Nine variants representing 0.27% of all reads presented indels only in the miR-200b RE. Eight variants (0.26% of all reads) presented edition of both miR-125b and -200b RE. The miR-125b target site was solely edited in 50 variants (4.7% of all reads).

To investigate if the same bias was observed in the edited transcripts, we used deep-sequencing to analyze the variations in the 3′UTR sequence of cScrt2 transcripts from CRISPR-edited embryos. From the 32.405 reads that were analyzed, 29.962 (92.4%) counts were non-variants and 2.443 (7.5%) were edited transcripts (Figure 4). Considering only the modified reads, the miR-125b RE was edited in 62% of them and the miR-200b RE was edited in 3.6%. Thus, these data indicate that most of the edited transcripts lacked the miR-125b target site. Further, it suggests that our data on CRISPR-mediated variation of Scrt2 expression is due to reduction of miR-125b action on Scrt2 transcripts.

## Discussion

Here we show evidence for the control of Scrt2 expression, during posterior neural tube development, by miRNAs miR-125b and -200b. We detected REs for these miRNAs in the 3′UTR of Scrt2 across vertebrates (zebrafish, chicken and 13 mammal species) and verified that their sites modulate expression, using a reporter assay in chick embryos. Moreover, the spatial expression pattern described for miR-125b and miR-200b (Darnell et al., 2006) is complementary to that of Scrt2, further supporting a possible modulation by miR-125b and -200b. Finally, ablating REs for both miRNAs with CRISPR/Cas9 or specifically inhibiting each miRNA activity with miRNA-125b sponge increased overall levels of cScrt2 and altered its expression pattern.

Although miRNAs can, rarely, act through the 5′UTR (Lytle et al., 2007), they typically effect post-transcriptional modulation through sites in the 3′UTR. We then transfected a chimera containing a luciferase reporter gene and the cScrt2 3′UTR region into chicken embryos (Miguez et al., 2013). The result was 68% less luciferase activity than the empty luciferase reporter, which indicates that the cScrt2 3′UTR region is indeed able to regulate expression, likely through miRNA action.

To test whether the reporter result was indeed due to the action of miR-125b or -200b (rather than through other miRNAs REs not identified in our analysis), and to ensure that miRNAs can modulate expression of cScrt2, we employed CRISPR/Cas9 to disrupt the miR-125b/-200b REs in the cScrt2 locus (Bhattacharya et al., 2018). This resulted in an increase of cScrt2 expression, detected with both RT-qPCR and *in situ* hybridization. This approach demonstrated a direct effect of miR-125b/-200b on cScrt2, in contrast with previous approaches, relying on miRNA inhibitors (reviewed by Zhang et al., 2013), which cannot rule out indirect effects, as the same miRNA may have several targets.

Because electroporating a two-parts system (Cas9 and sgRNA) resulted in a mosaic effect, with some cells receiving only one of the plasmids (Supplementary Figure S2), we FACS-selected Cas9-positive cells to quantify cScrt2 expression with RT-qPCR, in addition to *in situ* hybridization. Both methods showed increased cScrt2 expression, due to the disruption of miR-125b/-200b REs.

miR-125b REs ablation with CRISPR/Cas9 resulted in displacement of the cScrt2 expression pattern, with cScrt2-positive cells appearing in the proliferative zone (an inner layer of the neural tube) in addition to the IZ, which is the endogenous cScrt2 pattern (Vieceli et al., 2013). As cells move to more peripheral positions in the neural tube as they mature (Ashwell, 2009), the altered expression pattern suggests that cScrt2 transcription starts before what has been previously reported, but the mRNAs do not persist due to miR-125b/-200b action. In our CRISPR-edited gDNA we identified large deleted sequences and, as the 3′UTR is essential to the regulation of mRNA stability and translation efficiency (Barrett et al., 2012), large deletions in this portion could generate non-functional mRNAs, leading to degradation. The analysis of Scrt2 mRNA demonstrated the absence of large deletions at the 3′UTR and miR-125b-site changes in 62% of RE-edited transcripts. In other words, the majority of edited transcripts were lacking the miR-125b target site, suggesting that the phenotype generated was mostly due to decrease in miR-125b interference on Scrt2.

Further, the effect of the miR-125b sponge was stronger than that of miR-200b sponge, with a greater increase and displacement of cScrt2 expression in the neural tube. Considering that the absolute levels of miR-200b are lower than miR-125b, their titration by its sponge may have been more efficient than that of miR-125b. Together with the phenotypes generated by CRISPR-editing, these results suggest that miR-125b may be more relevant in controlling availability of cScrt2 transcripts.

Both miRNAs sponges in the DRG promoted an enhanced expression of cScrt2. This suggests that, similar to the neural tube, in the DRG these miRNAs could be controlling cScrt2 levels directly. Alternatively, since miR-125b/-200b represses genes related to EMT in neural crest cells (e.g., Snail1/2) (Gessert et al., 2010; Gradus et al., 2011; Shin et al., 2012), the ablation of miR-125b/-200b activity could result in an early or increased migration to form the DRGs, thus indirectly increasing the cScrt2-positive population in this tissue (del Barrio and Nieto, 2002).

The Scratch family modulates the expression of genes involved in cell adhesion and migration (Barrallo-Gimeno and Nieto, 2005; Paul et al., 2012; Itoh et al., 2013), and other members of the Snail superfamily, Snail1 and Snail2/Slug, are also involved in EMT and migration, through modulation of E-cadherin (Liu Y. N. et al., 2013; Villarejo et al., 2014). However, previous reports of miRNA-mediated modulation of Snail1 and Snail2/Slug are concentrated on metastatic EMT and embryonic stem cells. In various cancer cell lines, miR-200b reduces EMT through direct repression of Snail1 and Snail2/Slug (Burk et al., 2008; Gregory et al., 2008; Korpal and Kang, 2008; Gill et al., 2011; Kurashige et al., 2012; Liu Y. N. et al., 2013) and, in breast cancer, miR-125b promotes EMT through modulation of Snail and E-cadherin levels (Liu Z. et al., 2013; Nie et al., 2019). The IZ of the neural tube lacks Snail2 and Slug. Instead, Scratch2 is the member of the SNAIL superfamily that modulates EMT and cell migration in the anterior neural tube. In the cortex, Scrt1/2 promotes radial migration through repression of E-cadherin transcription (Itoh et al., 2013). The role of Scrt2 in cell migration at the posterior neural tube has not been shown yet. However, if Scrt2 does modulate cell migration, then miR-125b/-200b would also modulate EMT in this setting. Our data now shows that, in the posterior neural tube, miR-125b directly targets Scrt2, and thus could indirectly regulate the expression of Scrt2-target genes involved in EMT. Accordingly, the presence of miR-125b in the ventricular zone and outer layers (Darnell et al., 2006), indicate that they could suppress cell migration, whereas, in the IZ, where miR-125b levels are low, Scrt2 levels increase and centrifugal migration occurs. Together, these data suggest that miR-125b might control the cell migration through availability of Scrt2 transcripts. The evolutionary conservation of miR-125b and -200b REs at Scrt2 3′UTRs further suggests that this regulatory mechanism is conserved amongst vertebrates, and it may have arisen as a double-insurance mechanism during the evolution of Scratch2 gene in amniotes (Supplementary Figure S1). The miR-200b RE in Scrt2 is present in vertebrates but not lamprey whilst the miR-125b appears latter in amniotes.

The present work adds a new layer of knowledge concerning molecular neurogenesis, prompting the need for more research on the role of miRNAs during this process, and shedding light on the mechanisms behind the tight control of gene expression involved in the generation of cell diversity in the nervous system.

## Ethics Statement

The animal study was reviewed and approved by the Comissão de Ética no Uso de Animais (CEUA ICB/USP no. 025/2013).

## Author Contributions

FV conceived the study. CG performed the experiments and analyzed the results. CG, MS-C, and CY designed the experiments. SD and FV contributed with additional experiments and analysis. CG and CY wrote the manuscript. All authors contributed to the article and approved the submitted version.

## Conflict of Interest

The authors declare that the research was conducted in the absence of any commercial or financial relationships that could be construed as a potential conflict of interest.

## Footnotes

1 http://targetscan.org

2 http://www.geisha.arizona.edu

3 https://itol.embl.de

4 https://www.genscript.com/
