# Supplementary material for: Scratch2, a Snail Superfamily Member, Is Regulated by miR-125b
Source: Front Cell Dev Biol. 2020 Aug 25;8:769. doi: 10.3389/fcell.2020.00769 (PMC7477046; doi:10.3389/fcell.2020.00769)
Supplement: Supplementary file 1 [file Image_1.pdf]

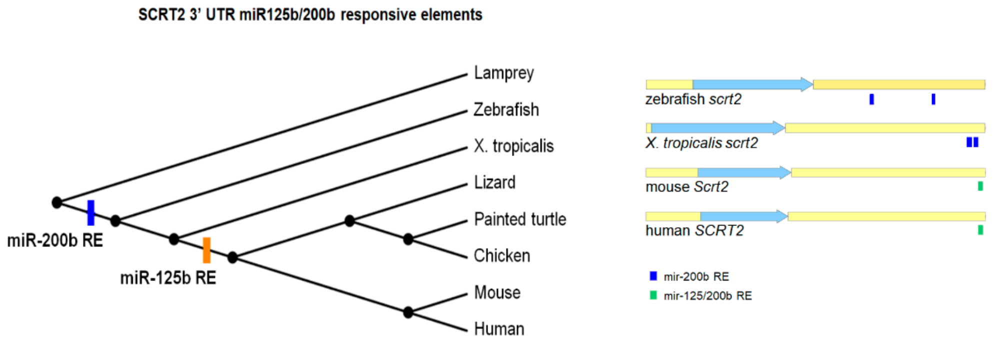

Supplementary Figure 1. Model for Evolution of the Responsive Elements in the Scrt2 gene. Simplified visualization of the vertebrate phylogenetic tree derived from multiple genome alignment available for download from the UCSC genome browser showing possible evolutionary points when target sites for miR-200b/125b targets sites on SCRT2 3' UTR may have been gained. The diagrams on the right show Scrt2 transcripts of different species with the relative position of UTRs, CDS and their miRNA responsive elements. The blue vertical bar indicates the miR-200b RE, the orange bar indicates the miR-125b RE and the green vertical bar indicates the presence of both MREs in the UTR.

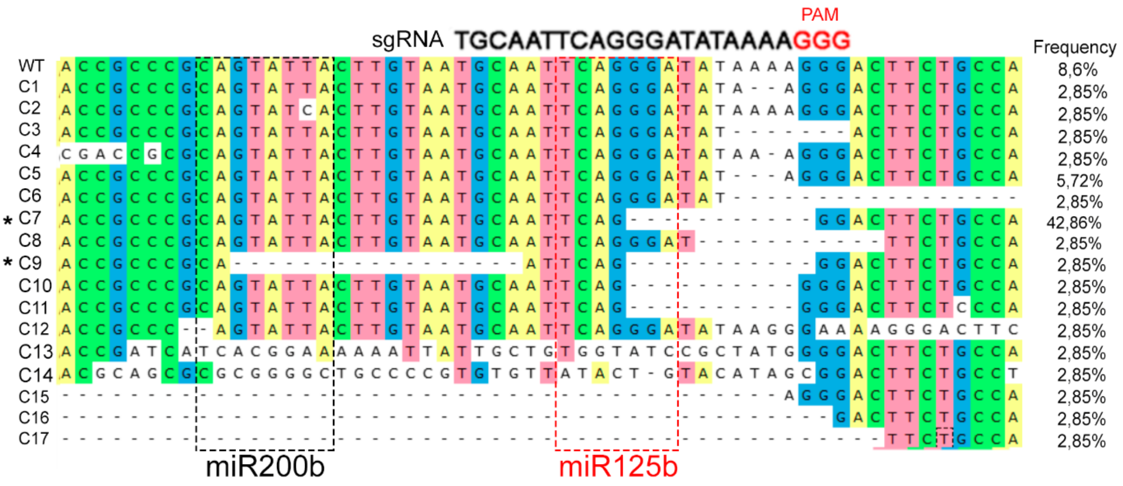

Supplementary Figure 2. Alignment of genomic cScrt2 3'-UTR terminal region (462-518 nt) after *in embryo* edition with CRISPR/Cas9 + sgRNA electroporation. Thirty-five clones were sequenced. WT, wild type (sequence similar to control cScrt2 3'-UTR) was detected in 8,6% of clones; C1 to C17 are some of the clone varieties found with their respective sequences and frequency. In around 23% of the clones, the miR-200b RE was edited, 20% of the clones had miR-125b RE edited and 14,25% lost both sites simultaneously. The black and red boxes indicate the seed sequences of miR-200b and -125b, respectively. The asterisk (*) indicates an edition that maintains miR-125b RE despite deletion of an intervening sequence.
